# How predictive of future healthcare utilisation and mortality is data-driven population segmentation based on healthcare utilisation and chronic condition comorbidity?

**DOI:** 10.1186/s12889-024-19065-w

**Published:** 2024-06-18

**Authors:** Andrea Gartner, Rhian Daniel, Ciarán Slyne, Kelechi Ebere Nnoaham

**Affiliations:** 1 Cwm Taf Morgannwg University Health Board, Ynysmeurig House, Navigation Park, Abercynon, CF45 4SN UK; 2 Division of Population Medicine, School of Medicine, Cardiff University, Cardiff, UK; 3 School of Medicine, Cardiff University, Cardiff, UK

**Keywords:** Population health, Population segmentation, Cluster analysis

## Abstract

**Background:**

In recent years data-driven population segmentation using cluster analyses of mainly health care utilisation data has been used as a proxy of future health care need. Chronic conditions patterns tended to be examined after segmentation but may be useful as a segmentation variable which, in combination with utilisation could indicate severity. These could further be of practical use to target specific clinical groups including for prevention. This study aimed to assess the ability of data-driven segmentation based on health care utilisation and comorbidities to predict future outcomes: Emergency admission, A&E attendance, GP practice contacts, and mortality.

**Methods:**

We analysed record-linked data for 412,997 patients registered with GP practices in 2018-19 in Cwm Taf Morgannwg University Health Board (CTM UHB) area within the Secure Anonymised Information Linkage (SAIL) Databank. We created 10 segments using k-means clustering based on utilisation (GP practice contacts, prescriptions, emergency and elective admissions, A&E and outpatients) and chronic condition counts for 2018 using different variable compositions to denote need. We assessed the characteristics of the segments. We employed a train/test scheme (80% training set) to compare logistic regression model predictions with observed outcomes on follow-up in 2019. We assessed the area under the ROC curve (AUC) for models with demographic variables, with and without the segments, as well as between segmentation implementations (with/without comorbidity and primary care data).

**Results:**

Adding the segments to the model with demographic covariates improved the prediction for all outcomes. For emergency admissions this increased discrimination from AUC 0.65 (CI 0.64–0.65) to 0.73 (CI 0.73–0.74). Models with the segments only performed nearly as well as the full models. Excluding comorbidity showed reduced predictive ability for mortality (similar otherwise) but most pronounced reduction when excluding all primary care variables.

**Conclusions:**

This shows that the segments have satisfactory predictive ability, even for varied outcomes and a broad range of events and conditions used in the segmentation. It suggests that the segments can be a useful tool in helping to identify specific groups of need to target with anticipatory care. Identification may be refined with selected diagnoses or more specialised tools such as risk stratification.

**Supplementary Information:**

The online version contains supplementary material available at 10.1186/s12889-024-19065-w.

## Background

Across the world, populations are ageing, and chronic conditions are increasingly prevalent, putting pressure on health care systems. There is growing recognition of the importance of patient-centred care combined with a population health approach that emphasises prevention and anticipatory care [1]. Population health management, which has population segmentation as a foundational core component, offers a practical approach to integrating person-centred care and population health.

Population segmentation involves grouping populations on the similarity of one or more proxies of health need and potentially allows definition of population groups that can be targeted with integrated and tailored health and care interventions [1]. There are two main types of segmentation approaches: expert-driven segmentation with a-priori defined criteria based on evidence review and expert-opinion, and data-driven segmentation using statistical methods to define the segments [2]. In recent years data-driven segmentation has been employed using cluster analyses of rich health care utilisation data as a proxy of future health care need [1, 3–5].

Two recent systematic reviews have identified several studies on segmentation. Some of these used expert-defined segments, different variables such as subsets of utilisation variables, and were based on utilisation in diverse settings, thereby making comparisons challenging, but we cite some relevant examples here [2, 5]. A study in England showed that utilisation-based segments had the potential to distinguish between patient groups with different care priorities [1]. It was successful in identifying lower needs populations but generalisability is a concern as it was based on a relatively affluent patient population and excluded A&E data [1]. A study in Singapore found that data-driven segmentation based on age and utilisation created clusters with specific health care needs in a longitudinal follow-up study of health care utilisation and mortality for the clusters [3]. The study did not include medication prescriptions, nor patients without a health care encounter in a single year, thereby excluding potentially healthier patients [3]. More recently, a study in the South Wales Valleys showed that utilisation-based segments, including A&E and prescriptions data, yielded groupings that distinguished between health and care needs but it did not include longitudinal follow-up [4].

Data-driven utilisation-based segmentation can generate segments that satisfy the statistical imperative of clustering by similarity on chosen attributes. However, it could also yield segments that lack clinical meaningfulness with respect to identifying practical opportunities for clinical intervention. Incorporating expert-knowledge of other clinically relevant population attributes into data-driven segmentation could theoretically enhance their performance. One such attribute is the individual burden of chronic conditions which, in traditional segmentation, has been shown to perform well in improving discrimination as well as providing a good indicator of health care spend [6]. The number of chronic conditions and increasing annual GP contacts predicted A&E attendance in England [7]. To our knowledge, comorbidity indicated by chronic condition counts has not been incorporated as a novel component into utilisation-based data-driven segmentation. Primary care data is not widely shared with secondary care providers or public health organisations, leading to interest in feasibility of using secondary care data only, such as in a study in Singapore that showed that a commercial segmentation tool could be employed using hospital data only [8].

Co-ordination, planning and support (including preventative care) for populations at greater risk of needing emergency care is a key plank of Welsh health policy [9] and segmentation based on past healthcare utilisation is a useful way of grouping people with broadly similar need to inform such co-ordination. In addition, of interest in our analyses is the ability of such utilisation-based segments, augmented with chronic condition comorbidity, to predict key outcomes (e.g., future emergency care use). We therefore set out to create generic data-driven segments that could (a) function as a proxy of need to help identify cohorts to target for intervention, and (b) be computationally deployed in practice to health professionals.

More specifically, the aims of this study were:

- To assess the predictive ability (specifically discrimination) of data-driven segmentation based on health care utilisation and comorbidity for a variety of outcomes: Emergency admission, A&E attendance, GP practice encounters and mortality.
- To investigate the extent to which including chronic condition counts or primary care data (GP practice contacts, prescriptions) in the segmentation variables could improve prediction of future healthcare need and mortality.

## Methods

### Data processing and cohort definition

We used record-linked data from the Secure Anonymised Information Linkage (SAIL) databank in Swansea University for 412,997 patients registered with a GP practice in Cwm Taf Morgannwg University Health Board (CTM UHB). This includes their GP and hospital activity data (including A&E) as well as the deprivation quintile using the Welsh Index of Multiple Deprivation 2019 [10]. We included patients who were registered with a GP practice CTM UHB at the end of 2018, having been registered in Wales throughout 2018 to fully capture their exposure and explore their outcomes in 2019. Patients who moved into Wales from England during 2018, for example, were excluded, as their records would be incomplete.

We calculated the number of emergency hospital admissions, elective admissions including day cases, A&E attendances and first and follow-up outpatient attendances during 2018 using hospital activity data. The first outpatient attendance is the start of the outpatient episode following a referral, the follow-up appointments subsequent appointments for the same episode. We estimated the number of GP practice contacts by counting the number of days any of a set of Read codes was found for each patient, indicating that a patient had been seen or contacted in person or by phone by a health professional. This set of Read codes was developed in collaboration with primary care. For prescriptions we chose to count the number of unique medications prescribed by a GP. We used codes from the Quality Outcomes Framework (QOF) version 38 [11], a widely used coding framework, to calculate whether patients had been diagnosed with a chronic condition since 2001 in the GP data. This version was chosen as the newest supporting Read code version 2 to match our data. If a condition was subsequently coded as resolved the patient was treated in our analysis as not having this condition. All listed QOF conditions were included: atrial fibrillation, asthma, cancer, coronary heart disease, chronic kidney disease, COPD, dementia, depression, diabetes, epilepsy, heart failure, hypertension, learning disability, psychosis or schizophrenia or bipolar, osteoporosis, non-haemorrhagic stroke, rheumatoid arthritis, transient ischaemic attack, stroke.

### Segmentation procedure

**Fig. 1:**
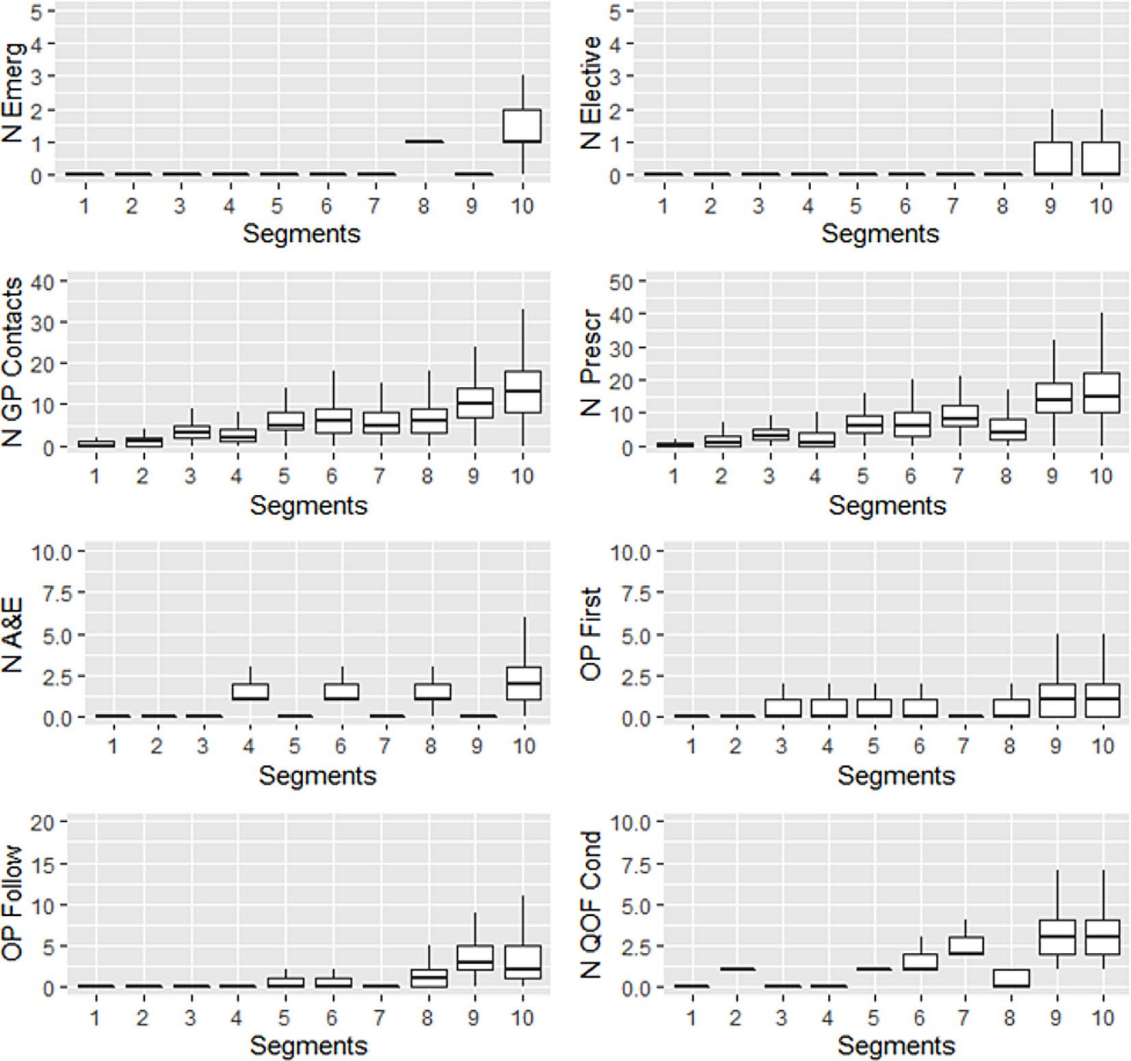
Box plots for segmentation variables

The data-driven segments were produced using the unsupervised k-means clustering method (based on squared Euclidean distances) using the ‘kmeans’ function from the ‘stats’ package in R [12]. The following patient-level variables were included (all as totals for 2018): the number of emergency hospital admissions, elective admissions, A&E attendances, GP practice contacts, unique prescriptions, first outpatient appointments, follow-up outpatient appointments and chronic conditions. As the results of k-means clustering are sensitive to outliers, we truncated the utilisation variables at specific values decided on clinical importance and based on inspecting the distributions of each variable (Emergency admission at 10, A&E at 20, GP Practice contacts, prescriptions, outpatients and elective admissions at 40 events). For example, we truncated the extreme frequent attenders to A&E found to commonly be classed as having 20 or more events per year [13]. Further information on truncation is included in Appendix A1. The values (truncated where needed) were log-transformed to deal with skewed data and scaled to between 0 and 1 to normalise [14]. We weighted the variables as follows: Emergency hospital admissions *2, A&E attendances *2, unique prescriptions *0.5, chronic conditions *2, all others were unweighted. These were initially chosen to distinguish by emergency admission and A&E to be able to target specific segments with interventions to reduce unscheduled care. Similarly, distinction on chronic condition counts could be useful to target those with comorbidities and particular utilisation patterns. We then examined the characteristics of resulting segments and selected the most promising weighting through this iterative process including expert clinical input on the weight. Small variations in weight did not show much difference and we did not further refine the weights. Unique medications were reduced by half to lower the importance of additional medications versus utilisation events. To decide on the number of segments we calculated the within-cluster sum of squares for different segment numbers [15], which was relatively low for ten segments (Figure A1 in the appendix) and were also considered a suitable number to practically implement whilst also giving sufficient nuance. We also checked that the segments captured the majority of the variation in each variable (Table A1 in Appendix A1), as measured by the percentage of total variance explained by the segments [16]. For ease of interpretation, we ordered the segments from low need Segment 1 to Segment 10 with the highest need in most settings by examining the descriptive characteristics, as shown in Fig. 1.

We compared the above main segments to two alternative segmentation procedures. The first excluded only chronic conditions (otherwise as above), thereby testing whether adding chronic conditions had improved the model. Similarly, the second alternative excluded primary care data, therefore only including hospital admissions, outpatients and A&E (otherwise as above). This model serves to assess whether only using secondary care data is feasible, where primary care data is not available.

### Descriptive analysis

To understand the characteristics we examined several variables by segment: the number of events of the utilisation variables, the number of chronic conditions, as well as demographic information on age, sex and deprivation. We inspected the distribution of the component variables of the segmentation using box plots. We also calculated the number of patients with the following outcomes in 2019: 1 or more emergency admissions, 2 or more A&E attendances, 5 or more GP practice contacts in 2019. Similarly, we calculated the percentage who died during 2019.

### Assessing predictive ability

To assess predictive ability more formally, a train/test scheme was employed to compare model predictions with the observed outcomes in the following year. This allowed an assessment of whether predictions based on the segments had an improved ability to predict future outcomes over those that did not include the segments, whilst avoiding so-called “over-fitting bias”.

We defined four outcome measures in 2019 for the logistic regression models: had 1 or more emergency admission, had 2 or more A&E attendances, had 5 or more GP practice contacts, and death. We classified the outcome as above/below mean utilisation on follow-up for emergency admissions (above = 1+, below = 0) and GP contacts (above = 5+, below = 0–4). For A&E attendances we chose to use 2 attendances as a cut-off rather than one (the mean is 0.32, Table 1) to reflect the range of severity and reasons for attendance, for example attendance with minor issues or reasons of proximity to A&E and lack of other accessible services. Two or more attendances were considered to capture recurring or more severe need.

The statistical measure used was the area under the ROC curve (AUC) [17]. One interpretation of the AUC is as the probability that a randomly chosen individual who experiences the outcome in the following year has a predicted risk that is higher than a randomly chosen individual who does not experience the outcome. As such, it is a measure of how well the model used for prediction distinguishes between those who will or will not experience the outcome. The data were split at random into a training set (80%) and a testing set (20%), with logistic regression models fitted using the former and the predictions and outcomes compared on the latter.

We analysed five different models to examine differences in the AUC when variables were added or excluded. The base Model A (demographic variables) included age group, sex and deprivation quintile. Model B included age group, sex, deprivation quintile as in Model A but also the main segments and serves to test whether the predictive ability of the segments does not merely reflect demographic variation amongst segments. Model C included only the main segments to test the predictive ability of the segments when used alone in practice. To also compare different segmentation methods, Model D used utilisation-only segments (excluding chronic conditions) to test whether adding chronic conditions had improved prediction and Model E used segments based on secondary care data (hospital admissions, A&E, outpatients) but excluding all primary care variables to test whether segments based on secondary care use alone could be implemented. We also assessed statistical significance of model differences using De Long’s method [18].

We also calculated the pseudo R^2^ of the logistic regression models using the training dataset as an estimate of model fit.

## Results

### Descriptive analysis

The main characteristics of each segment are summarised in Table 1 with box plots shown in Fig. 1. A description of characteristics of each segment is included in Appendix A1.

**Table 1:** Characteristics of patients by segment

	Segments										All
	1	2	3	4	5	6	7	8	9	10	segments
Demographics											
N	106,925	78,218	36,732	36,782	47,239	35,778	23,178	15,490	19,397	13,258	412,997
% of population	25.9	18.9	8.9	8.9	11.4	8.7	5.6	3.8	4.7	3.2	
Mean age	31.15	35.19	44.42	25.99	51.65	63.33	46.53	31.95	68.34	65.00	41.50
% Female	39.7	59.9	44.0	45.0	59.0	52.7	54.8	51.9	54.0	55.7	50.2
% in 40% most deprived	53.3	54.5	55.0	59.3	55.9	58.5	60.8	62.0	58.8	63.1	56.3
**Mean numbers of**:											
N GP contacts	0.39	4.18	1.00	3.17	6.29	6.00	6.78	6.75	11.38	14.51	4.14
N Prescriptions	0.46	3.72	2.01	2.52	6.98	9.19	7.56	5.52	15.33	16.72	4.71
N outpatients first	0.05	0.32	0.08	0.40	0.46	0.27	0.66	0.65	1.19	1.31	0.35
N Outpatients follow-up	0.11	0.68	0.20	0.63	1.07	0.37	1.17	1.73	4.34	4.14	0.85
N Emergency admissions	0.00	0.02	0.00	0.00	0.02	0.02	0.00	1.32	0.05	1.66	0.11
N Elective admissions	0.01	0.09	0.03	0.07	0.22	0.08	0.17	0.26	0.99	0.97	0.16
N A&E attendances	0.00	0.00	0.03	1.37	0.00	0.02	1.47	1.58	0.21	2.29	0.35
N chronic conditions	0.00	0.00	1.07	0.00	1.00	2.43	1.34	0.33	3.30	2.90	0.76
**Prevalence (%)**:											
Atrial Fibrillation	0.0	0.0	0.7	0.0	1.2	7.5	2.6	0.7	17.9	17.7	2.4
Asthma	0.0	0.0	27.9	0.0	16.3	23.9	26.9	6.4	22.7	21.5	9.9
Cancer	0.0	0.0	4.0	0.0	4.0	10.0	4.2	1.1	24.2	17.0	3.6
Coronary heart disease	0.0	0.0	1.7	0.0	2.2	13.8	3.9	1.3	24.9	22.8	3.8
Chronic kidney disease	0.0	0.0	1.5	0.0	1.5	14.9	3.2	0.4	26.1	21.0	3.7
COPD	0.0	0.0	1.0	0.0	2.2	10.5	3.3	1.0	17.1	17.3	2.8
Dementia	0.0	0.0	0.1	0.0	0.2	1.8	0.8	0.3	3.9	5.5	0.6
Anxiety, depression	0.0	0.0	40.9	0.0	33.0	40.3	45.1	12.4	37.4	36.9	16.9
Diabetes	0.0	0.0	2.3	0.0	6.8	28.7	8.4	1.8	36.9	27.1	6.6
Epilepsy	0.0	0.0	2.3	0.0	1.3	3.1	3.2	0.9	3.3	3.9	1.1
Heart Failure	0.0	0.0	0.2	0.0	0.2	3.1	0.6	0.1	8.8	8.5	1.0
Hypertension	0.2	0.3	19.2	0.2	26.7	62.1	23.8	5.3	65.8	51.5	16.5
Learning disability	0.0	0.0	1.1	0.0	0.6	1.3	1.2	0.2	0.8	1.2	0.4
Psychosis, schizophrenia, bipolar	0.0	0.0	1.3	0.0	0.6	3.3	1.9	0.4	4.3	4.7	0.9
Osteoporosis	0.0	0.0	1.2	0.0	1.7	6.2	2.4	0.4	11.8	9.3	1.8
Non-haemorrhagic stroke	0.0	0.0	0.1	0.0	0.0	2.4	0.3	0.0	4.6	4.9	0.6
Rheumatoid arthritis	0.0	0.0	0.5	0.0	0.9	1.7	0.9	0.2	5.4	2.9	0.7
Stroke	0.0	0.0	0.4	0.0	0.3	4.7	1.0	0.3	8.5	10.3	1.3
TIA	0.0	0.0	0.5	0.0	0.6	4.3	1.5	0.3	8.1	8.7	1.2

Table 2 shows the number of patients by 2018 segment with specific follow-up events in 2019. It shows broadly that those in higher need segments in 2018 (higher segment numbers) have higher crude percentages of patients with higher utilisation or death in 2019.

**Table 2:** Number of patients (crude percentages) with events in 2019 by segment in 2018

	Total patients in 2018	Patients with 1 + emergency admissions		Patients with 2 + A&E attendances		Patients with 5 + GP contacts		Patients who died	
Segment 1	106,925	3,100	(2.9%)	3,316	(3.1%)	6,599	(6.2%)	109	(0.1%)
Segment 2	78,218	4,643	(5.9%)	1,529	(5.5%)	22,654	(29%)	118	(0.2%)
Segment 3	36,732	1,695	(4.6%)	4,271	(4.2%)	6,616	(18%)	152	(0.4%)
Segment 4	36,782	2,836	(7.7%)	4,320	(11.7%)	8,487	(23.1%)	46	(0.1%)
Segment 5	47,239	3,768	(8%)	2,927	(6.2%)	24,348	(51.5%)	303	(0.6%)
Segment 6	23,178	2,897	(12.5%)	3,522	(15.2%)	12,690	(54.8%)	230	(1%)
Segment 7	35,778	4,039	(11.3%)	2,366	(6.6%)	21,418	(59.9%)	717	(2%)
Segment 8	15,490	3,014	(19.5%)	2,578	(16.6%)	7,009	(45.2%)	195	(1.3%)
Segment 9	19,397	4,394	(22.7%)	2,641	(13.6%)	15,963	(82.3%)	949	(4.9%)
Segment 10	13,258	5,252	(39.6%)	3,880	(29.3%)	10,804	(81.5%)	1,507	(11.4%)

Segments and total patients based on 2018 data, events on follow-up in 2019

We also compared the characteristics in 2018 segments with those created using 2019 data (data not shown). These were fairly similar suggesting some stability over time.

### Modelling analyses

For emergency admissions the full model (Model B: AUC 0.73, 95% CI 0.73–0.74) had better discrimination than the model with only demographic covariates (Model A: AUC 0.65, 95% CI 0.64–0.65). This was the case for all outcomes, suggesting that adding the segments to the model with demographic covariates had indeed improved the prediction (Table 3). This shows that the segments have discriminatory ability beyond the demographic information indirectly reflected within them.

Model C (AUC 0.71, 95% CI 0.71–0.72), which contained only the segments, discriminated nearly as well as the full model for emergency admissions. This pattern was similar for all outcomes, except for mortality where Model C was slightly worse than Model A and Model B. This model assesses the main segments that could be used in practical rollout.

Using utilisation-only segments and excluding comorbidity (Model D) led to similar discrimination to the main segments (Model C), except for mortality where discrimination was significantly lower (Table 3).

Model E, using the segments excluding all primary care variables, had lower discrimination than Model C, particularly for GP practice contacts and mortality.

The above patterns were also evident in the calculated Pseudo R^2^ values, shown in Table A3 in the Appendix.

**Table 3:** Ability to predict specific outcomes in the following year (AUC and 95% CI) compared for different covariates and different segments

	Emergency admissions	A&E attendance	GP practice contacts	All-cause mortality
N with outcome	35,638	31,350	133,070	4,327
N without outcome	377,359	381,647	279,927	408,670
Model A: with age, sex and deprivation	0.65 (0.64,0.65)	0.59 (0.59,0.6)	0.73 (0.73,0.73)	0.88 (0.88,0.9)
Model B: full model with segments (incl. chronic conditions), age, sex and deprivation	0.73 (0.73,0.74)	0.71 (0.7,0.72)	0.81 (0.81,0.81)	0.92 (0.92,0.93)
Model C: with segments (incl. chronic conditions) only	0.71 (0.71,0.72)	0.69 (0.69,0.7)	0.79 (0.79,0.79)	0.86 (0.85,0.88)
Model D: with utilisation-only segments (no chronic conditions)	0.71 (0.71,0.72)	0.69 (0.69,0.7)	0.78 (0.78,0.79)	0.79 (0.78,0.81)
Model E: with segments excluding primary care data	0.68 (0.67,0.69)	0.67 (0.66,0.67)	0.68 (0.68,0.68)	0.76 (0.74,0.78)

Emergency admission (0/1 + events), A&E (0 or 1/2 + attendances), GP encounters ( < = 4/5 + events); AUC from logistic regression trained on 80%, predicted on 20% of the data

There is no universally agreed classification of how well a model discriminates between two levels of an outcome in terms of exact thresholds and categories [19]. A value of 0.5 means no predictive ability (no better than chance), whereas a value of 1 means the model is perfectly able to distinguish between those who will and will not experience the outcome. General guidelines have been proposed by Hosmer et al.: AUC values of 0.7–0.8 as acceptable, 0.8–0.9 as excellent and 0.9-1 as outstanding discrimination [19]. Using this classification our full model ranged from acceptable to outstanding, whilst for segments only (Model C) it was classed as poor to excellent (A&E narrowly fell into the poor category). Overall, using the classification as a general guide only, we consider the segments to have achieved satisfactory discriminatory ability given the varied outcomes and broad range of events and conditions used in the segmentation.

## Discussion

This work set out to assess the predictive ability of our data-driven segmentation model and to determine whether including comorbidity or separately primary care data in the segmentation could improve prediction of future healthcare need. We found that adding the segments to the model with demographic covariates improved the models’ predictive ability for all outcomes, showing that the segments have discriminatory ability beyond the demographic information indirectly reflected within them. Models with the segments only were nearly as predictive as the full models, suggesting that the segments alone may be a useful practical tool in helping to identify specific groups of need whilst being feasible to practically implement in health care systems by, for example, matching patients’ data to the segments at regular intervals.

Comparisons to other studies are challenging as they tended to investigate health care costs or other outcomes than our study, used expert-defined segments, were set in other geographical regions, or used very different methods such as comparisons of survival time or regression model estimates [5, 20, 21]. One study in Singapore, using similar methods, investigated resource utilisation bands (RUB) from a commercial expert-driven segmentation model using very detailed clinical data [8]. Their results (AUC) for RUB groups of mortality (AUC 0.732), citing good discrimination, was lower than for our segments (AUC 0.86) [8]. Their full models including age and sex, and their best performing models using machine learning had lower discrimination than our full model (AUC 0.92) for mortality [8]. Whilst they used different study populations, adults only compared to all ages, but otherwise similar methods, it indicates that our segmentation model could be considered as having good or better discrimination for mortality.

Whilst there is no universally agreed classification of how well the model has predicted the outcomes in terms of exact thresholds, Hosmer et al. have proposed useful guidelines [19]. What is classed as a satisfactory AUC value also depends on the outcome, as reasons for A&E attendance and emergency admissions are complex. For example, outcomes such as those due to accidents or road traffic accidents could not reasonably be predicted from existing health data and an AUC value close to 1 would be too ambitious. Our aim was to develop broad generic segments that would discriminate sufficiently between groups experiencing and not experiencing a wide range of outcomes. This is different from, for example, developing a diagnostic tool aiming to predict a particular disease, which would typically require much higher AUC values to be deemed useful. Overall, we consider the segments to have achieved satisfactory predictive ability. As outlined, this is perhaps better than expected given the varied outcomes, and broad range of events and conditions used in the segmentation.

Whilst we used a data-driven segmentation with a k-means cluster analysis, we also included some expert input by determining a weighting, specifically weighting up emergency admission, A&E and the number of chronic conditions. This was initially chosen to provide specific distinction considering that reducing unscheduled care is an important goal for the health service. We also examined the characteristics of resulting segments and selected the most promising weighting through this iterative process. Also note that the segments were developed using the entire dataset rather than the training dataset, which could have resulted in slightly over-optimistic estimates of discrimination. However, as the outcome data on follow-up were not involved in the segment development the differences are likely to be very small. Another study comparing different segmentation methods favoured a locally calibrated decision tree over K-means cluster analysis [6]. We aimed to produce generic segments that were predictive for four different outcomes to measure need, but using a decision-tree would produce segments based on a single outcome, such as cost, rather than be used for several outcomes as in our study [16].

We also compared the above to two alternatives. Excluding comorbidity as a segmentation variable led to only worse performance for mortality but this was most pronounced when excluding all primary care variables. Primary care variables were particularly important for predicting GP practice contacts and mortality; excluding them led to substantially lowered discrimination. This is perhaps not surprising as patients with chronic conditions may be more likely to need ongoing primary care and prescription, and those with high GP practice contacts and prescriptions are likely to have higher mortality risk [22]. We therefore consider it important that primary care data is captured in the segmentation. We did expect that the addition of chronic conditions would improve the performance to a greater extent than we found, except for mortality. It is, however, likely that there is correlation with other variables, and that their specific need is already captured to some extent in GP practice contacts, prescriptions and secondary care utilisation. Both the number of chronic conditions and increasing GP contacts predicted A&E attendance in England, and may therefore be already captured [7]. In addition, not all conditions are included in our list and conditions are counted without consideration of severity, which could contribute to the relatively small improvement seen. Having noted that, we would suggest that it is of more practical use to have distinction of segments by the presence and number of chronic conditions, as this may allow the targeting of interventions and preventative care to segments with specific utilisation patterns, for example preventative care for patients with multiple conditions using only primary care to prevent exacerbation.

In this study we have used four different outcomes and for GP practice contacts and mortality the prediction was better than for emergency admission and A&E attendances. There is a large variety of reasons for emergency admission and particularly A&E attendance, for example accidents or maternity, which are less likely to be predictable using data on past health care utilisation or comorbidities. Proximity to A&E is likely to be another possible factor but was not included in our data. As we used the number of events, we may not have captured all of the severity and intensity of treatment, for example length of stay or procedures, for secondary care. Using event counts is, however, a practical solution to implementing data-driven segmentation of routine administrative data relatively simply even with limited processing power. Other data sources such as those relating to determinants of health (e.g. housing data) or social care data could be very valuable if available in future.

There are inevitably some limitations relating to the data. We had to estimate GP practice contacts from event Read codes as appointments data was not available. We therefore did not know which health professional had been seen and estimated the number of days with one or more codes that likely involved contact with a health professional in the practice (including by telephone and some online services). We may therefore have under- or overestimated the GP practice contacts depending on patterns of coding and how well the selected codes reflected this. We showed, however, that primary care data was important and improved predictive performance.

We used a large record-linked population-level dataset including activity data in primary and secondary care settings. This includes the population registered with GP practices in the area during 2018, including those who have not used health services. A study in Singapore, for example, included only those who had seen a health professional in the time period and potentially missed healthier patients [3]. We have, however, excluded those who have recently moved into the area from outside of Wales, to ensure a full year of data, or babies born during the year 2018. We expect that those over 1 year old are similar in profile to those already registered and are excluding some early activity of infants, but we expect this is unlikely to have changed the results substantially. We included public secondary care activity but as with most studies using administrative data sources, we have not captured private health care in our study. We followed individuals up during 2019, a single year, as we aimed to estimate need for the coming year with the view to repeat segmentation or at least assignment to segments regularly. There may be differences for certain conditions where greater need develops over several years that we would not have fully captured. Future work should include a longer follow up. We have considered our four outcomes in 2018/19, before the effects of Covid-19 on the health service and specifically utilisation, for example waiting lists [23]. Further work may be needed to assess the effect of the pandemic on the segmentation over time. Further work may also include assessing the predictive ability of the segments for specific age groups, for example adults or those aged 75 and over, as we have included all ages in our analysis. The segments are likely to be used in combination with other selection criteria in practice and may perform differently for those groups.

It is not clear how well our findings of predictive ability may be generalised to other areas. Our population lives in an area of higher deprivation compared to Wales overall (56.3% of patients live in the two most deprived quintiles, Table 1, compared to 40% in Wales) and includes some of the most deprived areas in Wales [10]. Our population also has a high burden of chronic disease and given this is a more weighted component of the segmentation it is likely that the segments would turn out differently in a generally less deprived or very homogenous population. Different weightings or number of segments could be employed to tailor our approach to other populations.

The main strength of the study is the study design, specifically the use of longitudinal modelling analysis allowing individual-level follow-up for four different outcomes in a relatively large population. We have also carefully considered the components of segmentation and have compared different predictive models to assess performance. We suggest that this work is a promising practical approach to segmentation to help identify groups with distinct future health care needs for targeting with interventions as well as planning of services.

## Conclusions

Overall, this statistical analysis shows that the segments have satisfactory predictive ability, specifically discrimination, for a variety of outcomes. For some this was perhaps better than expected given the varied nature of the outcomes and the broad utilisation events or conditions used to develop the segments. It suggests that they can be a useful tool in helping to identify specific groups at a higher risk. In practice, the segments will likely be coupled with other characteristics such as particular chronic conditions and more specialised tools such as risk stratification models developed specifically for particular outcomes.

### Electronic supplementary material

Below is the link to the electronic supplementary material.

Supplementary Material 1

## Data Availability

The datasets used in this study are available in the SAIL Databank at Swansea University, Swansea, UK, but as restrictions apply they are not publicly available. All proposals to use SAIL data are subject to review by an independent Information Governance Review Panel (IGRP). Before any data can be accessed, approval must be given by the IGRP. The IGRP gives careful consideration to each project to ensure proper and appropriate use of SAIL data. When access has been granted, it is gained through a privacy-protecting safe haven and remote access system referred to as the SAIL Gateway. SAIL has established an application process to be followed by anyone who would like to access data via SAIL at https://www.saildatabank.com/data/apply-to-work-with-the-data/. A full list of the Read codes used for the estimation of the GP contacts can be requested from the corresponding author.

## Abbreviations

A&E: Accident and Emergency
AUC: Area under the curve
COPD: Chronic obstructive pulmonary disease
CTM UHB: Cwm Taf Morgannwg University Health Board
SAIL: Secure Anonymised Information Linkage
TIA: Transient ischaemic attack
95% CI: 95% confidence interval
