# Supplementary material for: How predictive of future healthcare utilisation and mortality is data-driven population segmentation based on healthcare utilisation and chronic condition comorbidity?
Source: BMC Public Health. 2024 Jun 18;24:1621. doi: 10.1186/s12889-024-19065-w (PMC11184761; doi:10.1186/s12889-024-19065-w)
Supplement: Supplementary file 1 — Supplementary Material 1 [file 12889_2024_19065_MOESM1_ESM.docx]

**Appendix A**

**A1 Additional information on the segmentation method**

Figure A1 shows the sum of squares within a cluster, the so-called “elbow method” ^15^ which was relatively low for 10 clusters, being considered a suitable number to practically implement whilst also giving sufficient nuance. For example, whilst 16 segments would have given a lower sum of squares we felt that such a large number of segments would be difficult to describe and use in practice. We did also test segmentation for 6, 7, 8 and 9 segments and inspected the distributions of variables, but we decided these did not give sufficient nuance, for example not separating the high need groups into those with emergency care and those without.

**Figure A1: Sum of squares within a cluster for different numbers of clusters**


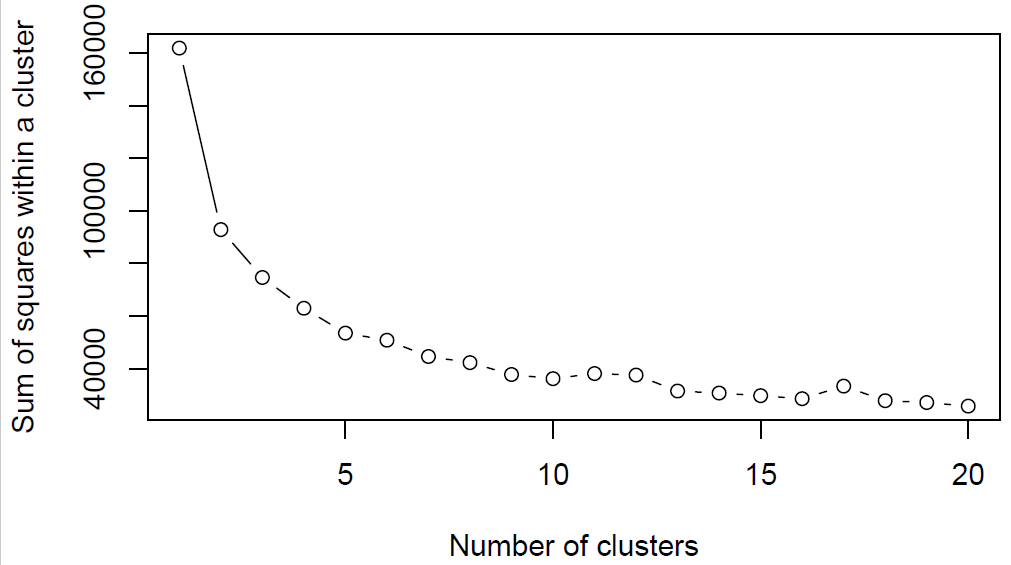


**Truncation of segmentation variables**

As the results of k-means clustering are sensitive to outliers, we truncated the utilisation variables at specific values decided on clinical importance and based on inspecting the distributions of each variable (Emergency admission at 10, A&E at 20, GP Practice contacts, prescriptions, outpatients and elective admissions at 40 events). For, example, A&E attendances of 20 or more per year are considered extreme frequent attenders ^13^ and thus precision of values above were not considered important for segmentation. A maximum of 0.11% of patients with events had the value truncated.

**Total variance explained by the segments**

We calculated, for each variable, the percentage of total variance explained by the segments (see Table A1). We calculated this by dividing the sum of the variances in each segment for each variable by the total variance for all patients for this variable * 100. The complement of the resulting percentage is the percentage explained by the segments ^16^. We checked that the segments explained the majority of variation in each variable.

**Table A1: Percentage of total variance for each variable explained by the segments**

| Chronic conditions | 82.4% |
| --- | --- |
| Emergency admissions | 63.8% |
| A&E attendances | 56.5% |
| Prescriptions | 55.3% |
| GP practice contacts | 49.8% |
| Outpatients first | 24.4% |
| Outpatients follow-up | 22.0% |
| Elective admissions | 6.1% |

**A2 Description of the segment characteristics**

A quarter of the population are in Segment 1, a generally younger segment without chronic conditions, and have very low utilisation on average. Patients in segment 2 also don’t have chronic conditions included in our list but these patients have utilisation of primary care and outpatients similar to the mean. Segment 3 includes patients who have a single chronic condition, particularly asthma and anxiety/depression, and generally have fewer GP practice contacts, prescriptions and utilisation in other settings. Patients in Segment 4 have low use except higher A&E attendances than average and do not have chronic conditions. Patients in Segment 5 have higher-than-average numbers of GP contacts and prescriptions, and one chronic condition, likely anxiety/depression, hypertension or asthma. Similar to Segment 5, patients in Segment 6 have higher-than-average GP contacts and prescriptions, only some outpatient attendances and 1-2 chronic conditions, with 45.1% having anxiety/depression. Patients in Segment 7, who are older and more deprived, have multiple chronic conditions, particularly anxiety/depression, diabetes and hypertension, and only increased GP practice contacts and prescriptions, suggesting they are managed in primary care. But it is also a much older and more deprived segment. Segment 8 includes much younger patients with higher utilisation than average, including higher A&E attendances, some in children. Segments 9 and 10 are the highest need segments including older patients on average with multiple chronic conditions, together making up 7% of the population. Segment 9 includes patients with very high utilisation but below average emergency care (emergency admissions and A&E attendances), whilst patients in Segment 10 have the highest levels of emergency care need.

**A3 Additional model results**

We calculated the statistical significance of the differences between models using De Long’s method implemented in R ^18^. The resulting p-values (shown in Table A2) have been considered in the reporting of any differences, for example the difference between Model D compared to Model C was only statistically significant for mortality. For the other outcomes we have referred to the models as having similar discrimination.

**Table A2: Comparison between models using De Long’s method, difference in AUC and statistical significance (p-value)**

|  | **Emergency admissions** | **A&E attendance** | **GP practice contacts** | **All-cause mortality** |
| --- | --- | --- | --- | --- |
| **Model B compared to Model A** | 0.08 ( <0.001) | 0.12 (<0.001) | 0.08 (<0.001) | 0.03 (<0.001) |
| **Model C: compared to Model B** | 0.06 (<0.001) | 0.1 (<0.001) | 0.06 (<0.001) | -0.02 (<0.001) |
| **Model C compared to Model A** | -0.02 (<0.001) | -0.02 (<0.001) | -0.02 (<0.001) | -0.06 (<0.001) |
| **Model D compared to Model C** | 0 (0.387) | 0 (0.475) | 0 (0.421) | -0.08 (<0.001) |
| **Model E compared to Model C** | -0.04 (<0.001) | -0.01 (0.02481) | -0.11 (<0.001) | -0.1 (<0.001) |

We also calculated the pseudo R^2^ of the logistic regression models (shown in Table A3) using the training dataset as an estimate of model fit.

**Table A3: Pseudo R^2^ for logistic regression models using the training dataset**

|  | **Emergency admissions** | **A&E attendance** | **GP practice contacts** | **All-cause mortality** |
| --- | --- | --- | --- | --- |
|  |  |  |  |  |
| **Model A: with age, sex and deprivation** | 0.05 | 0.02 | 0.11 | 0.23 |
| **Model B: full model with segments (incl. chronic conditions), age, sex and deprivation** | 0.11 | 0.08 | 0.22 | 0.29 |
| **Model C: with segments (incl. chronic conditions) only** | 0.09 | 0.07 | 0.20 | 0.20 |
| **Model D: with utilisation-only segments (no chronic conditions)** | 0.09 | 0.07 | 0.20 | 0.14 |
| **Model E: with segments excluding primary care data** | 0.07 | 0.06 | 0.08 | 0.11 |
